# The epidemiology and risk factors of chronic polyneuropathy

**DOI:** 10.1007/s10654-015-0094-6

**Published:** 2015-12-23

**Authors:** Rens Hanewinckel, Marieke van Oijen, M. Arfan Ikram, Pieter A. van Doorn

**Affiliations:** Department of Epidemiology, Erasmus University Medical Center, P.O. Box 2040, 3000 CA Rotterdam, The Netherlands; Department of Neurology, Erasmus University Medical Center, Rotterdam, The Netherlands; Department of Neurology, Alrijne Ziekenhuis, Leiderdorp, The Netherlands

**Keywords:** Polyneuropathy, Neuropathy, Epidemiology, Prevalence, Incidence, Idiopathic polyneuropathy

## Abstract

Polyneuropathy is a disabling condition of the peripheral nerves, characterized by symmetrical distal numbness and paresthesia, often accompanied with pain and weakness. Although the disease is often encountered in neurological clinics and is well known by physicians, incidence and prevalence rates are not well known. We searched EMBASE, Medline, Web-of-science, Cochrane, PubMed Publisher, and Google Scholar, for population-based studies investigating the prevalence of polyneuropathy and its risk factors. Out of 5119 papers, we identified 29 eligible studies, consisting of 11 door-to-door survey studies, 7 case–control studies and 11 cohort/database studies. Prevalence of polyneuropathy across these studies varies substantially. This can partly be explained by differences in assessment protocols and study populations. The overall prevalence of polyneuropathy in the general population seems around 1 % and rises to up to 7 % in the elderly. Polyneuropathy seemed more common in Western countries than in developing countries and there are indications that females are more often affected than males. Risk factor profiles differ across countries. In developing countries communicable diseases, like leprosy, are more common causes of neuropathy, whereas in Western countries especially diabetes, alcohol overconsumption, cytostatic drugs and cardiovascular disease are more commonly associated with polyneuropathy. In all studies a substantial proportion of polyneuropathy cases (20–30 %) remains idiopathic. Most of these studies have been performed over 15 years ago. More recent evidence suggests that the prevalence of polyneuropathy in the general population has increased over the years. Future research is necessary to confirm this increase in prevalence and to identify new and potentially modifiable risk factors.

**Electronic supplementary material:**

The online version of this article (doi:10.1007/s10654-015-0094-6) contains supplementary material, which is available to authorized users.

## Introduction

Polyneuropathy is a peripheral neuropathy characterized by symmetrical sensory symptoms, such as numbness, paresthesia and pain, and muscle weakness, which are predominantly located in the distal parts of arms and legs. Polyneuropathy is a disabling disease and has a negative impact on a person’s quality of life [1]. Although it is assumed that polyneuropathy affects a considerable proportion of the population, the exact prevalence and incidence of the disease are not well known. Elderly probably are at higher risk to develop polyneuropathy [2], and are thus at higher risk for associated falls and related injuries. Since an increasing proportion of the population is over 50 years of age, especially in developed countries, it is important to recognize the disease and to screen for treatable causes. Information about the frequency of the disease and its risk factors is therefore crucial.

Over 100 different causes of polyneuropathy have been identified, with diabetes as most important risk factor [2–6]. Guidelines have been developed to distinguish between these different causes [6–9]. Differentiation into acquired versus inherited, chronic versus acute and axonal versus demyelinating variants helps the diagnostic process in clinical practice. Most polyneuropathies have a progressive phase over months or years and have predominantly axonal characteristics with reduced sensory and motor nerve action potential amplitudes on electrophysiological examination [2]. However, even when diagnostic guidelines in patients with a slowly progressive axonal neuropathy are strictly applied, no cause can be identified in about 20–30 % of patients. These patients are often diagnosed with chronic idiopathic axonal neuropathy (CIAP) [10].

The aim of this review is to summarize the literature about the epidemiology of polyneuropathy and to obtain more information about differences across populations and between age groups. The review provides an overview of studies that investigated the prevalence and incidence of polyneuropathy and its associated risk factors.

## Methods

### Literature search

On January 8, 2015 (date last searched), we comprehensively searched the literature, using electronic medical databases (EMBASE, Medline, Web-of-science, Cochrane, PubMed Publisher and Google Scholar), to identify published studies reporting the prevalence or incidence of polyneuropathy in the general population. Our search strategy included a combination of terms about the disease of interest (polyneuropathy, peripheral neuropathy) and about epidemiology (epidemiology, prevalence, incidence). The specific search terms for each database can be found in the supplement. The search was limited to publications in the English language. We did not use a limitation for publication date. We initially selected publications that reported prevalence or incidence of peripheral neuropathy or polyneuropathy based on title and abstract. Studies that only investigated specific patients groups without a control group, for example only patients with diabetes, and studies that only investigated specific neuropathies, such as autonomic neuropathy, optic neuropathy, or mononeuropathy were not included. Studies about peripheral neuropathy were only included if the prevalence of polyneuropathy was also specified. When multiple articles from the same study were identified, the most recent or most comprehensive report was selected for this review. Our literature search was complemented by reviewing the reference lists of the identified articles, in order to gather other important publications that were missed with our search terms.

In addition to the prevalence of polyneuropathy in general, we further discuss some important risk factors for polyneuropathy and the prevalence of chronic idiopathic polyneuropathy. For this part of the review we also used hospital-based studies that specified risk factors like diabetes or intoxications. Therefore, we searched Medline for additional reports on frequency of different subtypes of polyneuropathy. We used the following search term: (neuropathy OR polyneuropathy OR neuropathies OR polyneuropathies) AND (workup OR diagnostic investigation OR cryptogenic OR idiopathic OR unspecified OR unclassified OR undetermined) and used the same limitations for this search as we did for the first one.

### Data collection

The following information was extracted from the selected studies: study size; geographical location (country); age distribution of the study population; screening protocol used; crude and, if available, standardized prevalence rates; age- and sex specific prevalence rates; incidence rates; cause-specific prevalence and, if possible, relative risks or odds ratios for risk factors of polyneuropathy.

## Results

**Fig. 1:**
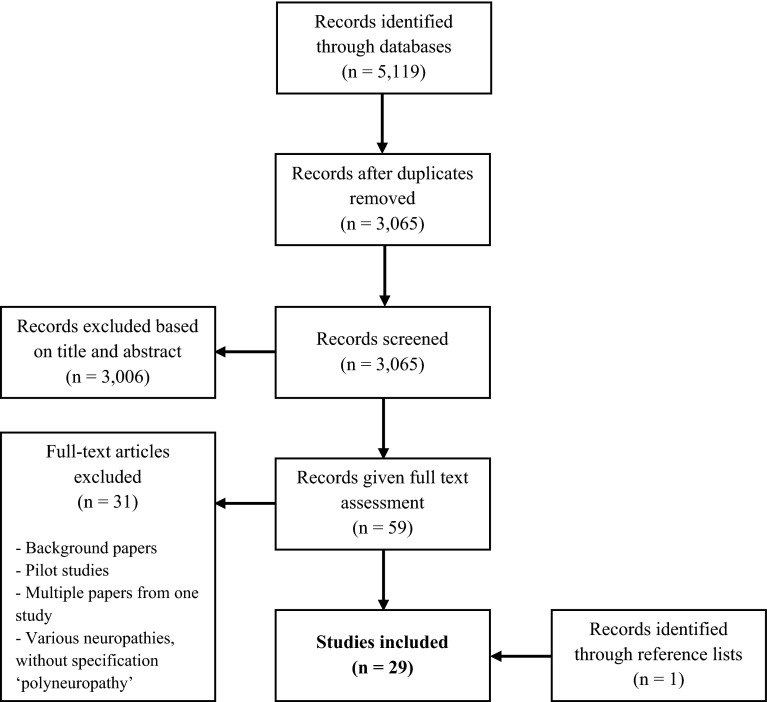
Selection of 29 studies that reported on the epidemiology of polyneuropathy

Our search yielded 5119 articles, of which 3065 were original articles. After excluding articles based on title or abstract, and after reading the full-text of the remaining articles, 28 studies remained. We included one additional reference that was identified after reviewing the reference lists of the selected articles. In total, 29 population-based studies that reported on the frequency of polyneuropathy were included in the review (Fig. 1). Twenty-eight studies reported the prevalence, but only three reported the incidence of polyneuropathy. One study only investigated the incidence of polyneuropathy. The studies were divided into three categories, based on study design: eleven door-to-door survey studies [11–21], seven case–control studies [22–28] and eleven cohort studies (seven cohort studies and four database studies) [29–39].

### Door-to-door survey studies (Table 1)

The World Health Organization (WHO) developed a protocol to study the epidemiology of major neurological disorders, which was specifically designed for developing countries where financial and medical resources are limited [40]. This protocol consists of two stages. In the first stage, a questionnaire to determine the presence of neurological symptoms and a brief examination to detect major neurological dysfunction are administered to the entire study population. This stage is often carried out by non-medical personnel (teachers, students, social workers) under supervision of a nurse or a neurologist. In screen-positive participants a neurologist performs a neurological examination to document the presence and type of the neurological disorder. The protocol includes screening for headache, epilepsy, stroke and peripheral neuropathy, among others. Peripheral neuropathy in this protocol includes mononeuropathies, radiculopathy and polyneuropathy. Only studies that specified the frequency of polyneuropathy cases were included in this review .

**Table 1:** Door-to-door survey studies reporting prevalence of polyneuropathy

Study/country/study year	Study size	Age of the study population	Assessment protocol	Prevalence of polyneuropathy	Prevalence of polyneuropathy related causes (per 1000)
Cruz et al. [11] Ecuador 1982	1113	All ages included; >50 years: 18 %	WHO protocol^a^	Crude: 9.0 per 1000	
Osuntokun et al. [12] Nigeria 1982–1983	18,954	All ages included; >50 years: 11 %	WHO protocol	Crude: 2.5 per 1000	1.9 tropical 0.4 idiopathic 0.1 diabetic 0.1 hereditary 0.1 nutritional
Cruz Gutierrez-del-Olmo et al. [13] Spain 1984	961	All ages included; >50 years: 30 %	WHO protocol^b^	Crude: 7.3 per 1000	3.1 idiopathic 2.1 diabetic 2.1 alcoholic
Longe and Osuntokun [14] Nigeria 1986	2925	All ages included; >50 years: 10 %	WHO protocol	Crude: 1.4 per 1000	
Bharucha et al. [15] India 1985	14,010	All ages included; >50 years: 44 %	Adapted WHO protocol	Crude: 7.1 per 1000	3.7 diabetic 2.1 idiopathic 0.4 toxic (alcohol and iatrogenic) 0.3 inflammatory 0.1 hereditary
Al Rajeh et al. [16] Saudi Arabia 1989	22,630	All ages included; >50 years: 4 %	Adapted WHO protocol	Crude: 0.8 per 1000	
Savettieri et al. [17] Italy 1993	14,540	All ages included; >40 years: 40 %	Adapted WHO protocol	Crude: 7 % screen positive^c^	2.1 diabetic
Lor et al. [18] Malaysia	100	Only subjects >65 years included	Bilateral distal symptoms and/or bilateral loss of pinprick or joint position sensation	Crude: 200 per 1000	
Kandil et al. [19] Egypt 1997	42,223	All ages included; >50 years: 10 %	Adapted WHO protocol	Crude: 8.3 per 1000	6.5 diabetic 0.9 idiopathic 0.5 metabolic^d^ 0.2 inflammatory 0.1 hereditary
Kruja et al. [20] Albania 2006–2008	9869	All ages included; >50 years: 31 %	≥2 symptoms + bilateral impairment of strength and/or sensation and/or reflexes with symmetrical distribution^e^	Crude: 32.5 per 1000 Adjusted^f^: 23.6 per 1000	
Dewhurst et al. [21] Tanzania 2009–2010	2232	Only subjects >70 years included	Self-developed two-phased screening tool. First phase based on questionnaire. Diagnosis according to WHO definition	Crude; 18.8 per 1000 Adjusted^f^: 18.6 per 1000	

Survey studies reporting prevalence of polyneuropathy. If reported, prevalence of polyneuropathy related causes is also shown

^a^WHO protocol: door-to-door screening with questionnaire and short examination, followed by a more comprehensive neurological examination performed by a neurologist to detect neurological disorders when screened positive in stage 1

^b^Protocol not specified, most likely WHO protocol

^c^Screening for all neuropathies, but only prevalence of diabetic neuropathy reported

^d^Including hypothyroidism, uremic and hepatic neuropathy

^e^Same protocol as Beghi et al. [29] (possible polyneuropathy criterion). Screening based on questionnaire, neurologist diagnosed polyneuropathy according to given definition

^f^Age-standardized to the WHO world standard population

Crude point prevalence of polyneuropathy in studies using this, or a similar protocol, ranged from 0.8 to 32.5 per 1000 (0.1–3.3 %) persons across all ages [11–17, 19, 20]. When only elderly are studied, prevalence ranges from 18.8 to 200 per 1000 persons (1.9–20 %) [18, 21]. There is a large variation in reported rates, but also in age distribution across different study populations, study area and study protocol (Table 1). Studies that report a low prevalence of polyneuropathy (0.8–2.5 per 1000) originate from African and Middle Eastern countries, such as Nigeria [12, 14], and Saudi Arabia [16]. In these studies only 4–11 % of the population is over the age of 50 years. In contrast, in European countries such as Spain [13], where polyneuropathy affects 7.3 per 1000 people, and in Albania [20], where polyneuropathy is reported in 32.5 per 1000 people, around 30 % is over 50 years of age. However, the latter study used a different assessment protocol and was performed 20 years after most of the other studies (Table 1).

Only two studies standardized the reported prevalence rates to a reference population [20, 21]. Adjusting the prevalence to the WHO world standard population resulted in an adjusted prevalence of 23.6 per 1000 (crude 32.5 per 1000) in Albania [20] and of 18.6 per 1000 (crude 18.8 per 1000) in Tanzania [21].

### Case–control studies (Table 2)

Seven reports compared the prevalence of polyneuropathy in patients with diabetes or prediabetes to a non-diabetic population-based sample of controls (Table 2) [22–28]. In four of these studies, persons known with diabetes or impaired glycemia were identified from medical databases and invited to participate in the study [22, 23, 25, 28]. A random sample of controls was selected from the same community [22, 25, 28] or practice [23] and matched to the diabetes patients on age [22, 23, 25, 28], sex [22, 23, 28] and ethnicity [22]. The three remaining case–control studies included participants from population-based surveys, where diabetes was assessed by self-report [24, 27] or by an oral glucose tolerance test [26]. Controls were randomly sampled from those without diabetes. Controls were categorized into (new) diabetes, impaired glucose tolerance, impaired fasting glucose or normal glycemia according to the results of an oral glucose tolerance test in four studies [22, 26–28].

**Table 2:** Case–control (survey) studies reporting prevalence of polyneuropathy

Study/country/study year	Selection of cases	Selection of controls	Number of participants	Assessment protocol	Definition of polyneuropathy	Prevalence of polyneuropathy
Franklin et al. [22] USA 1984–1986	Medical records from hospitals and physicians, and self-reports of persons aged 20–74 years	Random sample of households, matched on age, sex and ethnicity. Assessment with OGTT	DM: 277 IGT: 89 NGT: 486	Discomfort in the legs Reflexes Temperature sensation	≥2 abnormal items	DM: 25.8 % IGT: 11.2 % NGT: 3.5 %
Walters et al. [23] UK	Medical records from 10 practices. All > 30 years of age	Non-diabetics without glycosuria matched on practice, sex and birthdate.	DM: 1077 No DM: 480	Symptoms (numbness, burning, prickling, aching, tingling), light touch, pinprick, reflexes, vibration perception threshold (biothesiometer)	≥2 abnormal items	DM: 16.3 % No DM: 2.9 %
Harris et al. [24] Finland 1979–1981	National Health Interview Survey of people over 18 years. Self-reported diabetes	Random sample from those without diabetes	DM: 2829 No DM: 20,037	Numbness, pain or tingling, decreased ability to feel hot or cold	≥1 symptom	DM: 37.9 % No DM: males 9.8, females 11.8 %^a^
Partanen et al. [25] Finland 1979–1981	Newly diagnosed diabetes patients from district health centers, aged 45–64 years, Exclusion criteria: alcoholism, thyroid dysfunction, renal failure	Randomly selected controls without diabetes from the same age group, selected from population registry. Same exclusion criteria as cases	New DM: 132 No DM: 142	Symptoms: bilateral neuropathic pain, paresthesia Signs: atrophy, reflexes, touch, pinprick, vibration Nerve conduction velocity and amplitude in peroneal (4 values) and sural nerves (2 values)	Definite: ≥4 abnormal NCS values, including peroneal and sural nerve, and symptoms Probable: Same as definite but without symptoms, or one of the nerves involved with symptoms	Baseline:^b^ New DM: 8.3 % No DM: 2.1 % After 10 years: New DM: 41.9 % No DM: 5.8 %
Tapp et al. [26] Australia 1999–2000	AusDiab survey study of people >25 years of age. Assessment with OGTT to diagnose diabetes (and evaluation of current treatment)	Random sample of those with normoglycemia after OGTT	DM: 398 New DM: 423 IGT: 1009 IFG: 142 NGT: 464	Modified Neuropathy Symptoms Score (NSS) Modified Neuropathy Disability Score (NDS) Pressure perception test (PPT) with monofilament Postural blood pressure drop	≥2 of the scales abnormal (NSS > 4, NDS > 5, PPT < 6, fall in systolic blood pressure of ≥20 mmHg)	DM: 13.1 % New DM: 7.1 % IGT: 5.7 % IFG: 5.6 % NGT: 2.8 %
Ziegler et al. [27] Germany 1997–1998	Participants with self-reported diabetes from two surveys of the MONICA/KORA study, aged 24–74 years	Matched (age and sex) nondiabetic subjects were assessed with OGTT to determine glycemic status	DM: 195 IGT: 46 IFG: 71 NGT: 81	Michigan Neuropathy Screening Instrument (MNSI)	MNSI > 2	DM: 28.0 % IGT: 13.0 % IFG: 11.3 % NGT: 7.4 %
Dyck et al. [28] USA 2004	Patients known as having impaired glycemia were selected through databases and assessed with OGTT	Patients known as having a normal glucose, matched on age and sex, were assessed with OGTT	New DM: 218 IG: 174 NGT: 150	Neuropathy Symptoms and Change (NSC) Neuropathy Impairment Score (NIS) Composite scores of nerve conduction	Clinical judgment after abnormality in nerve conduction, NSC or NIS	New DM: 17.4 % IG: 12.6 % NGT: 12.7 %

Case–control studies reporting prevalence of polyneuropathy in patients with diabetes, prediabetes and a population-based control group

*OGTT* oral glucose tolerance test, *DM* diabetes mellitusl, *IGT* impaired glucose tolerance, *IFG* impaired fasting glucose, *NGT* normal glucose tolerance, *IG* impaired glycemia: IFG, IGT or impaired HbA1c

^a^Males 9.8 %, females 11.8 %. No numbers of total males and females are reported, average could not be calculated

^b^Probable and definite polyneuropathy are both considered polyneuropathy

These studies reported a crude prevalence of polyneuropathy in 7–42 % of patients with (newly diagnosed or known) diabetes, in 6–13 % of patients with prediabetes and in 2–13 % of controls. The main aim of these studies is to show whether prevalence of polyneuropathy varies across different stages of glycemic impairment and to determine which determinants are associated with polyneuropathy. Assessment methods, exclusion criteria and polyneuropathy definitions across these studies differ substantially (Table 2).

### Cohort studies (Table 3)

Three cohort studies also compared the prevalence of polyneuropathy in individuals with diabetes to individuals without diabetes [36–38]. However, in these studies all members from a specific community were invited before stratification on diabetes status, giving the opportunity to also assess prevalence of polyneuropathy in the whole population. In a study conducted in Canada, an adult population with a very high prevalence of diabetes (29 %) was investigated and an overall crude neuropathy prevalence of 7 % was reported [36]. Neuropathy was defined as loss of monofilament sensation at one or more sites on the feet in order to obtain a highly sensitive, but not very specific, screening tool. The two other studies were performed in China [37, 38]. Polyneuropathy was present in 13 % of adults of the She ethnic minority group of China [37] and in 4 % of the Han Chinese population over 25 years of age, free of renal failure or type 1 diabetes [38]. These studies used scoring systems (Toronto Clinical Neuropathy Scoring System and Neuropathy Symptom Score with Neuropathy Deficit Score respectively) to evaluate the presence of polyneuropathy (Table 3).

**Table 3:** Cohort studies reporting prevalence of polyneuropathy

Study/country/study year	Population	Age of the study population	Assessment protocol	Definition of polyneuropathy	Prevalence of polyneuropathy
Beghi et al. [29] Italy 1990–1993	4191 patients seen in GP’s office consultations for any reason	All > 55 years	Questionnaire followed by examination (strength, sensation, reflexes) when ≥ 2 symptoms	Possible: ≥1 abnormal item of exam Probable: ≥ 2 abnormal items	Crude: 3.6 %^a ^ Adjusted: 3.5 %^b^ Diabetes: 44 % Neoplasm: 10 % Alcohol: 6 %
Nakashima et al. [30] Japan 1991	Database of 7685 residents of Daisen Town	All ages included, about 45 % > 50 years	Medical records of hospitals, GPs and other sources	Not specified	Crude: 3.3 per 1000 Adjusted: 2.2 per 1000^c^
MacDonald et al. [31] Norway 1999	Database of 27,657 subjects from 3 GP practices in London	All ages included, about 28 % > 50 years	Medical records and notes from GPs and referral hospital	Clinical objective signs in the presence of an established cause, such as diabetes. Alternatively, an EMG diagnosis was required	Diabetes: Adjusted: 2 per 1000^d^ Other (excluding alcoholic): Adjusted: 1 per 1000^d^ Incidence^e^: Diabetes: adjusted: 0.5 per 1000/year^d^ Other: adjusted: 0.2 per 1000/year^d^
Mygland and Monstad [32] Norway 1999	Database of 155,464 inhabitants of Vest-Agder	All ages	Database of all patients with polyneuropathy referred to the only neurology center in the county	Clinically and electrophysiologically classified	Crude: 1.2 per 1000 Idiopathic: 26 % Diabetes: 19 % Hereditary: 12 % Alcohol: 10 % CIDP: 8 %
Mold et al. [33] USA 1999–2000	795 non-institutionalized subjects recruited from 9 GP practices	All > 65 years	Symptom questionnaire, fine touch, position and vibration sensation and ankle reflexes	1 or more complete bilateral peripheral neurologic deficits	Crude: 30.9 %
Eisen et al. [34] USA 1999–2001	1061 deployed and 1128 non-deployed Gulf war veterans	Mean age 31–33 years	Neurologic examination and nerve conduction studies	Idiopathic distal sensory, motor or sensorimotor polyneuropathy based on exam and/or NCS^f^	Crude: Deployed: 4.8 % Non deployed: 5.9 %
Baldereschi et al. [35] Italy 1992–1993	4500 participants of the Italian Longitudinal Study on Aging (ILSA): population-based cohort study	65–84 years	Screening: self-reported diagnosis, symptoms, ankle reflexes, heel gait, touch and pain sensation.	Full neurological exam, history and record review when positive on any of the screening items Diagnosis: clinical judgment	Crude: 7.4 % Adjusted: 7.0 %^g^ Diabetes: 39.2 % Idiopathic: 51.5 % Other: 9.3 % Incidence: 7.9 per 1000/year
Bruce et al. [36] Canada 2003	467 nonpregnant community members of the Sandy Bay First Nation	All > 18 years >50y: 18 %	10-g Monofilament on 10 sites of the foot	Unable to sense monofilament on one or more sites	Crude: 7.3 %
Lin et al. [37] China 2009	5385 subjects from the She population of China	All > 20 years, mean age 47 years	Toronto Clinical Neuropathy Scoring System (TCSS)	TCSS ≥ 6	Crude: 12.6 %
Lu et al. [38] China 2011–2012	2035 nonpregnant Han community members without type 1 diabetes or renal failure.	All > 25 years	Modified Neuropathy Deficit Score (NDS) and Neuropathy Symptom Score (NSS)	NDS ≥ 6, or NDS ≥ 3 and NSS ≥ 5	Crude: 4.0 %
Visser et al. [39] Netherlands 2010	Adult population of the province of Utrecht: 953,110	All ≥ 18 years	New cases that are registered in databases of all hospitals in the proximity of the province of Utrecht during a period of 1 year	Local guidelines: combination of symptoms and deficits compatible with polyneuropathy and diagnostic work-up for etiological diagnosis	Only incidence: Crude: 0.7/1000/year Adjusted: 0.5/1.000/year^h^ Diabetes: 32 % Idiopathic: 26 % Toxic: 14 % Immune-mediated: 9 %

Cohort studies reporting prevalence of polyneuropathy in a general population

^a^Average prevalence of probable polyneuropathy from two regions

^b^Age- and sex-standardized to the 1990 Italian population

^c^Age- and sex-standardized to the 1990 Japanese population

^d^Age- and sex-standardized to the 1991 United Kingdom population

^e^Incidence was calculated with data from 13 general practices, covering a population of 100,230 patients

^f^Only idiopathic or unexplained neuropathy. Alcohol abuse, HIV, hypothyroidism, diabetes and medication excluded

^g^Age-standardized to the 1992 Italian population

^h^Age-standardized to the WHO world standard population

In an effort to give a more precise population prevalence estimate of polyneuropathy, a large study in two Italian regions was conducted from 1990 to 1993. In this study 4191 subjects of 55 years and older, seen in General Practitioners’ office consultations for any reason, were investigated as a reflection of the general population [29]. Participants were screened with a 7-point yes/no screening questionnaire (muscle cramps, restless legs, burning feet, muscle pain, problems with object handling, impairment in standing and gait, and paresthesia). The questionnaire was pretested and validated in a hospital setting before initiation of the study. In this validation study sensitivity and specificity were 78 and 82 % respectively, using a cut-off of two positive answers. After two or more positive answers on the questionnaire, participants were examined by a neurologist for signs of polyneuropathy. Possible polyneuropathy (defined as neuropathic symptoms with bilateral impairment in at least one of the following modalities: strength, sensation or deep tendon reflexes) was present in 7.3 % of participants and probable polyneuropathy (symptoms and at least two abnormal modalities) in 3.6 % of participants. The age- and sex-adjusted prevalence rates for the two regions (adjusted to the 1990 Italian population) were 3.6 % for Varese and 3.3 % for San Giovanni Rotondo.

In the Italian Longitudinal Study on Aging (ILSA), a population-based cohort study, the prevalence of polyneuropathy was also investigated (Table 3) [35]. Participants were randomly included from eight municipalities, based on population registries (704 participants per municipality, 88 males and 88 females per 5-year age group; range 65–84 years). The polyneuropathy screening procedure consisted of an interview about symptoms (“have you ever had the feeling of burning pain and/or numbness, or paresthesia in the feet or legs”), a previous neuropathy diagnosis (“has a doctor ever told you that you suffer from neuropathy of the legs”) and drug treatments and of a brief neurological examination (heel gait, ankle tendon reflexes and touch and pain sensation), administered by a clinical investigator. Individuals with a self-reported diagnosis, at least one symptom, or at least one abnormal test on the examination underwent a clinical work up, which consisted of an evaluation of the medical history, an extensive neurological examination and a review of medical records. Nerve conduction studies and laboratory investigations were not part of the study protocol, but information about these measurements was extracted from medical records if available. The screening procedure had a sensitivity of 94.7 % and a specificity of 70 % in a pilot study of 20 cases and 20 controls. The ILSA study reported an adjusted prevalence of 7.0 % among 4500 participants aged 65–84 [35]. Three years after the baseline investigation, 2845 participants were screened for a second time with the same case-finding procedure. This yielded an incidence rate of 7.9 per 1000 person-years.

Other studies that are listed in Table 3 include four database studies [30–32, 39]. Two database studies used hospital registries to identify patients with polyneuropathy from a specific community [32, 39]. The other two additionally used medical records and notes from general practices [30, 31]. The diagnosis of polyneuropathy was based on the clinical picture, complemented with EMG according to local guidelines. In one study, no polyneuropathy definition was reported [30]. With this database approach, only registered cases are used to calculate prevalence or incidence rates, taking the whole population of the community as the denominator. The last two studies described in Table 3 include one general practitioner study assessing elderly with a less strict definition of polyneuropathy (at least one bilateral peripheral neurological deficit) [33], and one study investigating only idiopathic polyneuropathy in Gulf war veterans [34].

### Age and sex-specific prevalence across all studies

Studies that reported age-specific prevalence rates consistently showed a higher polyneuropathy prevalence in higher age categories of the studied population [12, 15, 17, 19, 20, 27, 29, 35, 38, 39]. Crude sex-specific prevalence rates are less consistent; most authors reported a higher prevalence in females [15, 17, 19, 20, 35, 36], with a ratio of 1.5–2:1. Two of these studies reported age-standardized, sex-specific prevalence rates and showed that this female predominance is not confounded by age [20, 35]. Other studies found no difference [27, 38], or a slight opposite result with a female:male ratio of about 1:1.4 [22, 39].

### Risk factors for chronic polyneuropathy

Several diseases and factors have been associated with polyneuropathy. Since polyneuropathy probably is a multifactorial disease, it is not entirely appropriate to attribute the development of polyneuropathy to only one factor. These factors should be considered as component causes, and not as one sufficient cause. For instance, not all patients with diabetes or alcoholism will develop polyneuropathy, so multiple (known and unknown) component causes probably contribute to the development of the disease [41]. In clinical practice often one factor or disease, such as diabetes or alcohol abuse, is considered as a main (sufficient) cause of polyneuropathy in an individual. Some of the aforementioned survey studies sub-classified polyneuropathy according to these different causes. Tropical neuropathies like leprosy are common causes of polyneuropathy in developing countries such as Nigeria, whereas diabetes is more common in countries or study populations with a higher socio-economic status like Italy, the Netherlands and Spain (Tables 1, 3). However, there is not much population-based data available.

Several investigators studied causes of polyneuropathy in hospital settings (Table 4) [32, 39, 42–48]. In all of these studies, diabetes is the most common cause of polyneuropathy, accounting for 18–49 % of all cases. Other known important causes of polyneuropathy include alcohol abuse, toxic agents, such as chemotherapeutic drugs, nutritional deficiencies, immune-mediated causes and hereditary factors. Despite laboratory investigations, the cause in patients with a chronic axonal polyneuropathy cannot be identified in 12–49 %. Although there are probably some differences in the etiology of these polyneuropathy subtypes, it is likely that they share multiple common etiological factors. Investigation of risk factors in specific subtypes is therefore also important for polyneuropathy in general. Some of the most common conditions related to polyneuropathy and chronic idiopathic axonal polyneuropathy will be discussed briefly.

**Table 4:** Hospital-based studies investigated causes of polyneuropathy

Study	George and Twomey [42]	Lin et al. [43]	Johannsen et al. [44]	Mygland et al. [32]	Verghese et al. [45]		Rosenberg et al. [46]	Vrancken et al. [47]	Rudolph and Farbu [48]	Visser et al. [39]
Country	UK	Taiwan	Denmark	Norway	USA		Netherlands	Netherlands	Norway	Netherlands
Study period	1980–1984	1988–1989	1993–1999	1999	1990–1999		1993–1997	1999–2002	2000–2005	2010
Population	Patients referred to hospital for NCS	Patients seen in 5 neurological centers	Patients referred to hospital for NCS	Patients referred to hospital neurologist	Patients referred to EMG laboratory		Patients at outpatient department	Multicenter study of patients with a diagnostic work-up for PNP	Patients referred to hospital neurologist	New hospital-registered cases
Number of patients	74	520	147	192	231	171	172	137	226	743
Age	≥65	–	18–70	–	65–75	≥75	26–93	≥18	9–92	≥18
*Associated risk factor* (%)										
Cryptogenic/CIAP	28	12	25	26	13	27	20	49^e^	28	26
Diabetes	27	49	32	19	46	31	38	26	18	32
Malignancy	13	2	1	4	3	4	1	3	3	
Inflammatory	11	8	2	8	7	4	1	4	16	9^f^
Toxic medication	4	3	5	6	7	6	5	3	–	14 ^g^
Connective tissue disorder/vasculitis	4	–	3	5	1	2	1	4	4	5
Nutritional deficiency^a^	4	1	2	4	1	1	1	9	4	3
Alcohol	3	9	19	10	6	1	9	6	10	14 ^g^
Renal failure	3	4	1	–	2	2	4	4	–	4 ^h^
Hereditary	1	4	1	12	7	8	3	7	14	5
Sarcoidosis	1	–	–	2	–	–	1	–	–	–
Hypothyroidism	–	2	–	–	1	1	1	3	4	4 ^h^
Ischemic	–	2	1	–	0	2	–	–	–	–
Paraproteinemia	–	2	5	4	1	4	1	9	–	–
Liver disease	–	1	–	–	–	–	–	–	1	–
Infection^b^	–	–	1	1	0	1	1	–	2	–
Critical illness	–	–	1	–	3	4	1	–	–	–
HIV	–	–	1	–	–	–	12^d^	–	–	–
Other causes	–	2	3^c^	–	2	3	–	–	–	–

^a^Including vitamin B1 and vitamin B12 deficiency

^b^Infection includes borrelia infection, leprosy and other unspecified infections

^c^2 % unspecified metabolic disorder. Thyroid dysfunction not reported

^d^HIV referral center

^e^Largest center is a CIAP referral center

^f^Inflammatory neuropathies in this study not only include GBS and CIDP, but also polyneuropathies associated with paraproteinemia, paraneoplastic antibodies/malignancy and HIV-associated neuropathy

^g^Toxic medication and alcohol abuse are combined in this study and accounts for 14 %

^h^Thyroid dysfunction and renal function are combined in this study and accounts for 4 %

### Diabetic polyneuropathy

Prevalence of diabetes is 6.4 % worldwide and this number is expected to rise the next decades [49]. Diabetes can lead to several types of peripheral neuropathy, such as distal symmetric polyneuropathy, autonomic neuropathy, mononeuropathy and non-compressive radiculopathy. Polyneuropathy is the most common presentation [50]. The Italian General Practitioner Study Group reported a relative risk of polyneuropathy associated with diabetes of 8.8 (95 % confidence interval 6.1–12.8) [51]. Polyneuropathy occurs in up to 50 % of patients with diabetes and diabetes accounts for 18–49 % of all polyneuropathy cases (Table 4). Sensory symptoms are usually more prominent than motor involvement and neuropathic pain is a common disabling symptom, occurring in 40–60 % of patients with diabetic neuropathy [50]. Diabetic polyneuropathy has an axonal subtype in most cases. Treatment is mainly symptomatic. Potential modifiable risk factors associated with neuropathy in patients with diabetes include dyslipidemia, hypertension and obesity [50, 52–56]. Whether these factors also contribute to the development of polyneuropathy in non-diabetic subjects remains to be verified.

### Alcoholic polyneuropathy

Polyneuropathy is reported to be present in 13–66 % of chronic alcoholics, depending on diagnostic criteria used to diagnose neuropathy [57, 58]. The relative risk of polyneuropathy in chronic alcoholics is estimated at 3.9 (95 % confidence interval 1.5–9.0) [51]. There has been debate whether neuropathy in alcoholics occurs due to direct toxic effects of ethanol, due to a secondary thiamine deficiency or due to a failure of tissues to utilize thiamine in the presence of alcohol [57, 58]. Both alcoholic neuropathy and thiamine-deficiency neuropathy are mainly of the axonal type and are usually characterized by (painful) sensory disturbance and weakness in the distal parts of the lower extremities. Autonomic dysfunction often occurs. There is accumulating evidence that there are differences in the clinical phenotype between alcoholic neuropathy and thiamine-deficiency neuropathy. Pure alcoholic neuropathy without accompanying thiamine deficiency mainly affects small fibers, leading to slowly progressive sensory-dominant symptoms, neuropathic pain and impaired superficial sensation, whereas thiamine-deficiency neuropathy predominantly affects large fibers, leading to a more progressive, or even acute, polyneuropathy with predominantly motor symptoms [57, 58]. Since alcohol abuse often coexists with nutritional deficiencies, combined small and large fiber polyneuropathies are frequently found. Treatment, other than alcohol cessation and improvement of nutritional intake, is symptomatic.

### Hereditary polyneuropathy

Hereditary motor and sensory neuropathy, also called Charcot–Marie–Tooth disease (CMT) is the most common form of inherited peripheral neuropathy. CMT has an estimated prevalence of 40–82 per 100,000 people [59, 60]. Mutations in genes encoding major structural proteins of myelin, axonal transport and mitochondrial metabolism have been described [60]. These gene mutations ultimately lead to slowly progressive weakness, wasting and sensory symptoms in distal body parts, starting at the feet. These patients usually have high arches, hammer toes and weakness and wasting of intrinsic muscles of the feet that will progress in the lower legs in later stages of the disease. There are demyelinating (CMT1, CMT3 and CMT4), axonal (CMT2) and mixed or intermediate (CMTX and dominant intermediate CMT) types of CMT. Age of onset, severity and type of symptoms, family history, presence of other neurological signs (such as involvement of the central nervous system), and especially nerve conduction studies can give clues to determine the specific subtype and possibly involved genes. No specific treatment is currently available [59].

### Inflammatory neuropathies

Inflammatory neuropathies are reported in 2–16 % of all polyneuropathy cases depending on the clinical setting of the study (Table 4). Inflammatory neuropathies can present as a rapidly progressive sensorimotor polyneuropathy with a nadir within 4 weeks, known as the Guillain–Barre syndrome [61] and as a more chronic, relapsing-remitting or gradually progressive polyneuropathy that develops over a period of more than 8 weeks, as in chronic inflammatory demyelinating polyradiculoneuropathy (CIDP) [62].

CIDP is the most common chronic acquired demyelinating polyneuropathy. Prevalence rates vary between 1 and 7 per 100,000 people, but this may be an underestimation since the clinical presentation can be rather diverse, leading to under diagnosis [62]. CIDP likely has an autoimmune origin and is a treatable disorder. Patients can be treated with intravenous immunoglobulins, steroids or plasma exchange [62, 63].

### Other causes

There are many more factors, such as vitamin B1 or B12 deficiency, paraproteins, connective tissue disorders (systemic lupus erythematosus, Sjögren’s syndrome) and toxic agents (like chemotherapy) that are associated with polyneuropathy. When patients over the age of 50 have a slowly progressive symmetrical axonal polyneuropathy and no cause can be established, these individuals are usually diagnosed as chronic idiopathic axonal polyneuropathy (CIAP) [64–68].

### Chronic idiopathic axonal polyneuropathy

CIAP occurs in 12–49 % of polyneuropathy cases (Table 4), depending on the clinical setting (secondary versus tertiary center, or referral center for specific diseases). Precise population-based prevalence estimates are lacking. A recent population-based database study from the Netherlands reported that 26 % of incident polyneuropathy cases were idiopathic. An incidence rate of 30.3/100,000 person-years for persons 40 years or older was found [39].

CIAP is characterized by an insidious onset of symptoms usually starting in the sixth decade or later, and seems to affect males more than females [10, 39, 64, 69]. Symptoms are predominantly sensory, characterized by distal loss of sensation (pain, numbness and tingling), with or without weakness. The legs are more affected than the arms and distribution is usually symmetrical. The disease is slowly progressive and most patients remain ambulatory with mild to moderate disability, but all patients experience a reduced quality of life. Neurological examination shows decreased or loss of vibration sense, diminished perception of pain and light touch in a stocking like distribution and ankle reflexes are often absent [64, 70]. Electrophysiological examination shows features of an axonal polyneuropathy, usually with reduced or absent sensory nerve action potentials of the sural nerves and decreased amplitudes of the peroneal compound motor action potential [64, 70]. Quantitative sensory testing may show abnormal temperature and vibration thresholds [70]. Diagnostic criteria have been developed to improve recognition and diagnosis of CIAP [70].

CIAP probably constitutes of a heterogeneous group of conditions. Current research suggests a role for the metabolic syndrome, which includes impaired glucose tolerance, dyslipidemia, hypertension and obesity [65]. Studies showed that the metabolic syndrome is an independent risk factors for macro- and microvascular complications such as retinopathy, nephropathy and neuropathy in patients with diabetes [54, 71, 72]. Studies also showed that the metabolic syndrome is more prevalent in patients with CIAP [65, 73]. Impaired glucose metabolism probably is the most important factor attributing to the development of polyneuropathy, although results are not entirely consistent. Independent associations with dyslipidemia and obesity have also been reported [22, 27, 28, 65, 68, 73–80]. It is likely however that yet undiscovered factors also contribute to the development of CIAP.

## Discussion

We identified 29 population-based studies that investigated the epidemiology of polyneuropathy. There is a large variation in reported prevalence rates across these studies (0.1–12.6 % across all ages, 1.9–30.9 % in elderly), which is probably due to the diversity in assessment protocols, definition of polyneuropathy, study populations and study designs. Many studies rely on a two-step screening protocol. Participants are screened with a questionnaire, sometimes in combination with a short neurological examination, and only screen-positive participants are examined by a trained physician, usually a neurologist. In order to get a valid estimate of the prevalence of a disease, this first stage should identify all cases as screen-positive (sensitivity should be 100 %). A low number of screen positive participants without disease (high specificity) is also preferred, especially when resources and time are limited. Studies that do not use a two-step approach, but only use symptoms or signs, or a combination of both into a component score as diagnostic protocol need to be both sensitive and specific in order to obtain a valid estimate of the prevalence.

Most information is derived from door-to-door survey studies. An advantage of these studies is that similar research protocols have been used in large study populations and that the diagnostics can be done with relatively few resources. These studies give insight in the epidemiology of several neurological disorders, but may underestimate the prevalence of polyneuropathy, since subclinical polyneuropathy can be missed and refusal to participate in the study may give rise to selection bias. As these studies were not primarily focused on polyneuropathy and did not include an extensive neuropathy work-up, including nerve conduction studies, the results highly depend on the sensitivity of the screening procedure in the first stage, which is often not optimal. Despite this, most studies report a high sensitivity for the entire screening protocol. Overall, prevalence of polyneuropathy in door-to-door survey studies from developed countries seems higher than in studies performed in developing countries. This may partly be explained by a larger proportion of elderly people included in studies from developed countries. Standardizing prevalence to the same reference population is helpful to investigate this confounding effect of age, but unfortunately not many studies have standardized their prevalence rates. Other reasons for this variation can be differences in genetic, socioeconomic and environmental factors and differences in prevalence of associated risk factors for neuropathy. For example, alcohol consumption is considered to be less common in most developing countries [81], and prevalence of diabetes is lower, especially in Africa [49].

The case–control studies that were identified were primarily focused on determining an association between diabetes, prediabetes and neuropathy. Although these case–control studies give an estimate of the occurrence of non-diabetic polyneuropathy in controls, they are not suitable to give a population prevalence of polyneuropathy, because the distribution of cases and controls likely differs from the general population. Although three other studies included all inhabitants from a specific community [36–38] before stratifying for diabetes, the assessment methods (with low sensitivity or low specificity), exclusion criteria or low participation rate, indicate that the population prevalence estimates are most likely overestimated or biased.

The four database studies that investigated the frequency of polyneuropathy probably all underestimate the true incidence or prevalence, since only previously diagnosed patients were identified in these studies. Symptomatic individuals who do not visit a doctor, asymptomatic individuals, and individuals not being referred to a hospital (in case of hospital-based database studies) because there is a clear cause for the complaints (e.g. diabetes) are missed with this approach. The cohort study performed by the Italian General Practitioner Study Group was one of the first extensive community studies specifically designed to investigate polyneuropathy in an unselected elderly population. A ‘probable’ neuropathy was present in 4 % and a ‘possible’ polyneuropathy was diagnosed in 7 % of the participants who visited their general practitioner [29]. The results found in this study might lack validity due to selection bias. On the one hand, patients who visit a general practitioner may be less healthy and at a higher risk for polyneuropathy, due to chronic diseases or medication use, leading to an overestimated prevalence rate. On the other hand, some persons who have an increased risk to develop neuropathy, such as alcoholics or severely impaired patients, might be less likely to visit a general practitioner, leading to an underestimation of the prevalence. An unselected sample of 93 patients from the same general practitioners was visited and assessed at home. In this small sample, probable polyneuropathy was present in 4.3 %. This suggest a modest underestimation in the screened population (3.6 %). However, prevalence might also be underestimated, because only symptomatic patients were included in the study and sensitivity of the screening instrument was only 78 %. Moreover, nerve conduction studies were not performed.

The ILSA study reported a prevalence of polyneuropathy in persons over 65 years of age of 7 % [35]. Participants were randomly selected from database registries, probably leading to an unbiased and random sample of the general population. The case-finding procedure had a desirably high sensitivity and did not only rely on symptoms. This probably resulted in the most unbiased and reliable estimate of the prevalence of chronic polyneuropathy in the general elderly population. However, nerve conduction studies were not part of the study protocol and no polyneuropathy work-up, including laboratory investigations, was performed. Therefore, detailed information about causes and subtypes of polyneuropathy was not available.

Both these cohort studies reported a polyneuropathy prevalence of around 7 % [29, 35],which is much higher than the rates found in the door-to-door surveys, which are close to 1 % [13]. In the two Italian cohorts only elderly were included and the screening protocols were primarily focused on the detection of polyneuropathy, whereas most survey studies screened for a variety of neurological disorders across all ages. This might explain the higher prevalence found in these cohort studies.

Almost all before mentioned studies, including the ILSA study, were performed fifteen to 20 years ago. Since that time, life-expectancy, the proportion of elderly in the population and prevalence of obesity and diabetes increased [49, 82]. Perhaps this resulted in an increase in the incidence of polyneuropathy as well, which is also suggested by the results of the survey study performed in Albania from 2006 to 2008 [20]. This study reported a polyneuropathy prevalence of 3 % in the total general population (including all age categories), using a similar screening method as the Italian General Practitioner Study Group. Whether polyneuropathy is truly more prevalent than it was 20 years ago has to be confirmed in properly designed, large population-based studies.

## Conclusions and future directions

Prevalence of polyneuropathy in the general population ranges from 1 to 3 % and increases to 7 % in the elderly. Prevalence seems to depend on socioeconomic status and the age distribution of the study population. In developing countries the prevalence is lower, which can possibly be explained by a smaller proportion of elderly in the population and by differences in the prevalence of polyneuropathy risk factors. Life-expectancy and prevalence of associated risk factors have increased in the last decades. Whether this resulted in more patients with polyneuropathy is yet unknown. There is a need for more, properly designed, large studies that investigate the prevalence and risk factors of polyneuropathy in the general population. A cohort study of a general, unselected population would be the most ideal study design to give an unbiased estimate of the prevalence and incidence of polyneuropathy. Population surveys may also be used, but in general, available data and case definitions in these studies are less detailed than in cohort studies. To assess risk factors for polyneuropathy, case–control studies may be more efficient than cohort studies, but may also be more prone to biases. Heterogeneity in polyneuropathy definitions in past studies makes comparison between studies difficult. To overcome this, future studies should use a similar definition and screening protocol for polyneuropathy. Unfortunately, a gold standard test for polyneuropathy does not exist. A combination of neuropathic symptoms, neuropathic signs and abnormal nerve conduction studies provides the most accurate diagnosis of polyneuropathy. Therefore investigating prevalence of polyneuropathy in a large population is challenging. Ideally, new studies should uniformly include all these three aspects [7]. Standardizing results to a reference population is encouraged in order to ease comparison between studies.

Hopefully, future large prospective cohort studies that assess the presence of chronic diseases together with cardiovascular, metabolic, hereditary and lifestyle factors will also focus on disorders of the peripheral nervous system. These studies should also incorporate the assessment of polyneuropathy both cross-sectionally and longitudinally during follow-up over the years. This will hopefully give insight into new risk factors for this disabling condition.

## Electronic supplementary material

Supplementary material 1 (PDF 14 kb)
