# Supplementary material for: The epidemiology and risk factors of chronic polyneuropathy
Source: Eur J Epidemiol. 2015 Dec 23;31:5–20. doi: 10.1007/s10654-015-0094-6 (PMC4756033; doi:10.1007/s10654-015-0094-6)
Supplement: Supplementary file 1 — Supplementary material 1 (PDF 14 kb) [file 10654_2015_94_MOESM1_ESM.pdf]

The epidemiology and risk factors of chronic polyneuropathy

R. Hanewinckel MD[1,2], M. van Oijen MD PhD[1,2,3], M.A. Ikram MD PhD[1], P.A. van Doorn MD PhD[2].

[1]Department of Epidemiology, Erasmus University Medical Center, Rotterdam, The Netherlands

[2]Department of Neurology, Erasmus University Medical Center, Rotterdam, The Netherlands

[3]Department of Neurology, Alrijne Ziekenhuis, Leiderdorp, The Netherlands

Corresponding author: M.A. Ikram, MD PhD

Department of Epidemiology, Erasmus University Medical Center

P.O. box 2040, 3000 CA, Rotterdam, The Netherlands

Tel: +31 10 7043489 / Fax: +31 10 7044657 / E-mail: m.a.ikram@erasmusmc.nl

Supplementary data

Search terms for different databases. Date last search 08-01-2015

Embase.com 2007
(polyneuropathy/exp OR neuropathy/de OR 'demyelinating neuropathy'/exp OR 'peripheral neuropathy'/de OR 'diabetic neuropathy'/de OR 'sensorimotor neuropathy'/exp OR 'motor neuropathy'/exp OR 'nerve conduction disorder'/de OR 'sensory neuropathy'/exp OR (polyneuropath* OR polyradiculoneuropath* OR polyradioneurit* OR ((poly OR multiple OR peripheral OR amyloid OR 'giant axonal' OR uremic OR alcohol OR diabet* OR demyelinat* OR idiopath* OR cryptogen* OR sensorimotor* OR motor* OR sensor* OR 'distal symmetric') NEAR/3 (neuropath* OR polyneurit* OR neurit*)) OR ('nerve conduction' NEAR/3 (disorder* OR defect* OR deficit* OR disturb*)) OR ((neuropathy OR neuropathies) NOT ((optic* OR auditor*) NEAR/3 neuropath*))):ab,ti) AND (incidence/exp OR prevalence/de OR 'epidemiological data'/de OR (incidenc* OR prevalen* OR (epidemiolog* NEAR/3 (data OR monitor* OR assess*))):ab,ti) AND ('community assessment'/de OR 'community sample'/de OR community/de OR population/de OR 'urban population'/de OR 'rural population'/de OR 'population research'/de OR 'population based case control study'/de OR (population* OR communit*):ab,ti) AND [english]/lim NOT ([Conference Abstract]/lim OR [Letter]/lim OR [Note]/lim OR [Conference Paper]/lim OR [Editorial]/lim)

Medline (ovidSP) 1376
(exp polyneuropathies/ OR Diabetic Neuropathies/ OR (polyneuropath* OR polyradiculoneuropath* OR polyradioneurit* OR ((poly OR multiple OR peripheral OR amyloid OR giant axonal OR uremic OR alcohol OR diabet* OR demyelinat* OR idiopath* OR cryptogen* OR sensorimotor* OR motor* OR sensor* OR distal symmetric) ADJ3 (neuropath* OR polyneurit* OR neurit*)) OR (nerve conduction ADJ3 (disorder* OR defect* OR deficit* OR disturb*)) OR ((neuropathy OR neuropathies) NOT ((optic* OR auditor*) ADJ3 neuropath*))).ab,ti.) AND (incidence/ OR prevalence/ OR Epidemiological Monitoring/ OR Epidemiologic Measurements/ OR (incidenc* OR prevalen* OR (epidemiolog* ADJ3 (data OR monitor* OR assess*))).ab,ti.) AND (exp population/ OR (population* OR communit*).ab,ti.) AND english.la. NOT (letter OR news OR comment OR editorial OR congresses OR abstracts).pt.

Cochrane 55
((polyneuropath* OR polyradiculoneuropath* OR polyradioneurit* OR ((poly OR multiple OR peripheral OR amyloid OR 'giant axonal' OR uremic OR alcohol OR diabet* OR demyelinat* OR idiopath* OR cryptogen* OR sensorimotor* OR motor* OR sensor* OR 'distal symmetric') NEAR/3 (neuropath* OR polyneurit* OR neurit*)) OR ('nerve conduction' NEAR/3 (disorder* OR defect* OR deficit* OR disturb*)) OR ((neuropathy OR neuropathies) NOT ((optic* OR auditor*) NEAR/3 neuropath*))):ab,ti) AND ((incidenc* OR prevalen* OR (epidemiolog* NEAR/3 (data OR monitor* OR assess*))):ab,ti) AND ((population* OR communit*):ab,ti)

Web-of-science 1445
TS=(((polyneuropath* OR polyradiculoneuropath* OR polyradioneurit* OR ((poly OR multiple OR peripheral OR amyloid OR "giant axonal" OR uremic OR alcohol OR diabet* OR demyelinat* OR idiopath* OR cryptogen* OR sensorimotor* OR motor* OR sensor* OR "distal symmetric") NEAR/3 (neuropath* OR polyneurit* OR neurit*)) OR ("nerve conduction" NEAR/3 (disorder* OR defect* OR deficit* OR disturb*)) OR ((neuropathy OR neuropathies) NOT ((optic* OR auditor*) NEAR/3 neuropath*)))) AND ((incidenc* OR prevalen* OR (epidemiolog* NEAR/3 (data OR monitor* OR assess*)))) AND ((population* OR communit*))) AND LA=(english) AND DT=(Article)

PubMed publisher 36
((polyneuropath*[tiab] OR polyradiculoneuropath*[tiab] OR polyradioneurit*[tiab] OR ((poly[tiab] OR multiple[tiab] OR peripheral[tiab] OR amyloid[tiab] OR giant axonal*[tiab] OR uremic[tiab] OR

alcohol[tiab] OR diabet*[tiab] OR demyelinat*[tiab] OR idiopath*[tiab] OR cryptogen*[tiab] OR sensorimotor*[tiab] OR motor*[tiab] OR sensor*[tiab] OR distal symmetric*[tiab]) AND (neuropath*[tiab] OR polyneurit*[tiab] OR neurit*[tiab])) OR nerve conduction disorder*[tiab] OR nerve conduction defect*[tiab] OR nerve conduction deficit*[tiab] OR nerve conduction disturb*[tiab] OR ((neuropathy[tiab] OR neuropathies[tiab]) NOT ((optic*[tiab] OR auditor*[tiab]) AND neuropath*[tiab])))) AND ((incidenc*[tiab] OR prevalen*[tiab] OR (epidemiolog*[tiab] AND (data[tiab] OR monitor*[tiab] OR assess*[tiab])))) AND ((population*[tiab] OR communit*[tiab])) AND english[la] AND publisher[sb]

Google Scholar 200

Polyneuropathy|"peripheral|diabetic neuropathy" incidence|prevalence|"epidemiological data|monitoring|assessment" community|population
